# Partnering with community-based organizations to improve equitable access to depression care for underserved older adults in the U.S.: Qualitative formative research

**DOI:** 10.3389/fpubh.2022.1079082

**Published:** 2023-01-30

**Authors:** Lesley E. Steinman, Amanda T. Parrish, Marlana J. Kohn, Sherry Wu, KeliAnne K. Hara-Hubbard, Lori Brown, Syed Imam, Barbara Baquero, Peggy A. Hannon, Mark B. Snowden

**Affiliations:** ^1^Health Promotion Research Center, Department of Health Systems and Population Health, University of Washington, Seattle, WA, United States; ^2^Southeast Washington Aging and Long-Term Care, Yakima, WA, United States; ^3^Union for Pan Asian Communities (UPAC) Positive Solutions Program, San Diego, CA, United States; ^4^Department of Psychiatry and Behavioral Sciences, University of Washington, Seattle, WA, United States

**Keywords:** older adults, equity, implementation, dissemination, depression, community-based organizations

## Abstract

**Background:**

Embedding evidenced-based programs (EBPs) like PEARLS outside clinical settings can help reduce inequities in access to depression care. Trusted community-based organizations (CBOs) reach older adults who are underserved; however, PEARLS adoption has been limited. Implementation science has tried to close this know-do gap, however a more intentional focus on equity is needed to engage CBOs. We partnered with CBOs to better understand their resources and needs in order to design more equitable dissemination and implementation (D&I) strategies to support PEARLS adoption.

**Methods:**

We conducted 39 interviews with 24 current and potential adopter organizations and other partners (February–September 2020). CBOs were purposively sampled for region, type, and priority older populations experiencing poverty (communities of color, linguistically diverse, rural). Using a social marketing framework, our guide explored barriers, benefits and process for PEARLS adoption; CBO capacities and needs; PEARLS acceptability and adaptations; and preferred communication channels. During COVID-19, interviews also addressed remote PEARLS delivery and changes in priorities. We conducted thematic analysis of transcripts using the rapid framework method to describe the needs and priorities of older adults who are underserved and the CBOs that engage them, and strategies, collaborations, and adaptations to integrate depression care in these contexts.

**Results:**

During COVID-19, older adults relied on CBO support for basic needs such as food and housing. Isolation and depression were also urgent issues within communities, yet stigma remained for both late-life depression and depression care. CBOs wanted EBPs with cultural flexibility, stable funding, accessible training, staff investment, and fit with staff and community needs and priorities. Findings guided new dissemination strategies to better communicate how PEARLS is appropriate for organizations that engage older adults who are underserved, and what program components are core and what are adaptable to better align with organizations and communities. New implementation strategies will support organizational capacity-building through training and technical assistance, and matchmaking for funding and clinical support.

**Discussion:**

Findings support CBOs as appropriate depression care providers for older adults who are underserved, and suggest changes to communications and resources to better fit EBPs with the resources and needs of organizations and older adults. We are currently partnering with organizations in California and Washington to evaluate whether and how these D&I strategies increase equitable access to PEARLS for older adults who are underserved.

## 1. Introduction

Depression is a major public health issue for older adults—a leading cause of disability, poor function, increased morbidity, suicide and other mortality, and reduced quality of life (1). Late-life depression often goes unrecognized or undertreated for older adults (2), and older adults facing inequitable access to care include communities of color (3–6), linguistically diverse (6, 7), experiencing poverty (8), or in rural areas (9, 10), recognizing that many older adults are multiply marginalized by intersecting identities (11). The burden of late-life depression was exacerbated during the COVID-19 pandemic due to isolation, distancing, fear, and reduced access to services and supports (12).

Community-based social service organizations (CBOs) offer an important avenue toward increasing access to depression care for older adults who are underserved (13). CBOs reach these older adults (14), who often prefer non-pharmacological treatment delivered by trusted providers in their community (15–17). These organizations address unmet social needs, thus reducing obstacles to health and health care and lowering preventable differences in depression burden among marginalized older adults (13, 18). Improving access to quality housing, food, environments, and health care is a key strategy for health equity—for everyone to have a fair and just opportunity to be as healthy as possible. Likewise, global mental health researchers and practitioners call for closing the mental health care gap by building capacity among CBO providers who are typically non-clinical workers. Although historically these organizations have not been mental health care providers, they are uniquely positioned to reduce inequities in access to depression care by providing services in resource-constrained settings (19).

One model for community-based depression care is the Program to Encourage Active and Rewarding Lives (PEARLS) (20, 21). Although PEARLS was created with community partners, uptake has been limited (22). This “know-do gap” (23, 24) has been well-defined by the implementation science field, which has recently called for centering equity as the key indicator of success (25) so that efforts to increase reach do not exacerbate inequities. To date, we have used a diffusion model (26) of disseminating PEARLS through trained providers and organizations, local, state and national conferences and meetings, and inclusion in program repositories. While intended to spread PEARLS through naturally occurring networks, this passive dissemination approach may in fact disenfranchise resource-constrained CBOs by favoring organizations that already have training, funding, and serve English-speaking clients in urban areas (27). Just as some older adults' face health inequities, many CBOs that engage older adults experience resource scarcity.

The intent of this study was to partner with organizations that engage older adults at higher risk for depression, experiencing poverty, and with limited access to care (communities of color, linguistically diverse, and/or living in rural areas), to learn how we can more equitably disseminate and implement PEARLS with, for, and in these settings. Proactive partnership- and capacity-building with these CBOs has the potential to increase access to PEARLS depression care in their communities. This formative research is the essential first step of our efforts to design new PEARLS dissemination and implementation strategies that will reach new CBOs and improve equity in access to care.

## 2. Methods

We are a CDC-funded prevention research center—a community-academic partnership based at a public university school of public health that collaborates with clinical and community partners to translate research into policy and practice to promote health equity. In late 2019, we received a 5-year grant to reduce inequities in access to depression care for older adults. This manuscript describes the first step in this study: conducting formative research with organizations that engage older adults who are underserved in order to develop equity-centered dissemination and implementation (D&I) strategies that align with CBO strengths and needs. This study is guided by a social marketing framework—an approach to promoting organizational and provider behavior change for social good (28). Social marketing can enhance pull factors like increasing organization's motivations to adopt PEARLS by understanding potential adopters through market or audience research, communicating how much potential adopting organizations can influence PEARLS through appropriate channels that target different populations, taking the social system into account (e.g., networks and norms), and ongoing engagement and evaluation with the target audience (29). This framework aligns with health equity and community engagement approaches like community-based participatory research (CBPR) and designing for dissemination (30, 31). These research strategies emphasize good communication between researchers and community partners, partnership exchange of knowledge, skills and resources guided by mutually understood values and through different means, community capacity building, and collaborations that benefit both researchers and community partners (32). This study was approved by the University of Washington Institutional Review Board in January 2020.

### 2.1. PEARLS intervention

The Program to Encourage Active and Rewarding Lives (PEARLS) is an evidence-based program for late-life depression care developed in partnership between our research center and CBOs. PEARLS reaches older adults traditionally underserved by clinical care by providing the program *via* CBOs that are already offering accessible services and supports. In six-to-eight 1-h visits over a 5-month period, trained CBO staff (“coaches”) meet one-on-one with participants and help them build problem-solving skills using Problem Solving Treatment (PST) (33) to gain a sense of control over issues in their lives that are overwhelming. Participants also use Behavioral Activation (BA) (34) to plan meaningful activities that are physical, social, and pleasant, learn about depression to address stigma and understand symptoms, and link to health and social supports as needed. By training front-line social service staff in a structured intervention coupled with regular clinical supervision, PEARLS uses task shifting (35) from specialty or clinical mental health providers to expand access to depression care.

In 2004, our community-academic partnership co-developed and tested PEARLS *via* a randomized controlled trial (RCT) with CBOs and older adults who were underserved (42% persons of color, 58% annual income < $10,000, 72% lived alone, average of 4–5 chronic conditions) and lacked access to mental health care (9% receiving mental health care in last 6-months) (20). PEARLS participants were three times as likely to improve their depression outcomes as older adults in usual care. Since then, our research center has supported PEARLS delivery through training and technical assistance to foster our community of practice. The Guide to Community Preventive Services and the Administration on Community Living now recommend PEARLS to treat depression in older adults (21). As of 2021, PEARLS has reached over 9,400 older adults through 133 CBOs in 26 states.

### 2.2. Participants

Our participants are organizations in Washington and California that engage older adults who are underserved. For this project, we prioritized the following populations experiencing poverty who are underserved by depression care: older adults of color, who are linguistically diverse (speak languages other than English), and/or live in rural areas. While there are different national and local financial resources to support PEARLS delivery, we selected these two states because they have well-defined funding mechanisms to support PEARLS adoption-a key implementation strategy for feasibility and sustainability (36). We recruited three types of organizations that could fund, deliver, or support PEARLS implementation (funders, CBOs, other partners), sampling both organizations that have adopted and not adopted PEARLS (current adopters and potential adopters, respectively). Table 1 provides further detail about the different types of organizations in our sample. We used maximum variation purposive sampling (37) at the organization level to engage decision-makers (e.g., directors) and “do-ers” (e.g., community health workers) at these organizations. All participating organizations engaged our priority older populations.

**Table 1:** Defining organizations for interview sampling.

**Organization**	**Definition**
Funders	Local government agencies with available funding for PEARLS • California: county mental health departments with state Mental Health Services Act funding • Washington: Area Agencies on Aging with access to state Medicaid waiver funding
Community-based organizations (CBOs)	Community-based social service organizations that can deliver PEARLS (e.g., culturally-specific organizations and senior centers)
Other partners	Organizations that make up care systems but would not directly fund or deliver PEARLS (e.g., faith-based organizations, community-based clinics, food safety-net organizations, and state social service agencies).
Current adopters	Funders or CBOs currently funding or delivering PEARLS
Potential adopters	Funders or CBOs working in communities who are underserved and not currently funding or delivering PEARLS

### 2.3. Data collection

Qualitative research methods yield rich, contextual data about complex organizational and social phenomena (38). We conducted semi-structured interviews to describe in-depth processes, realities, and experiences from multiple perspectives (39). Our interview guide asked about the context for PEARLS adoption (e.g., words to talk about depression, whether depression is a priority for organization or community, perceived stigma around depression); barriers and benefits to PEARLS adoption (e.g., fit, cultural appropriateness, value); organizational capacity and needs (e.g., how services are provided before and during COVID-19, how our center can better support them); and potential collaborators and competitors for PEARLS adoption (e.g., collaborators for screening, referrals, funding, and support; other alternatives to PEARLS). For PEARLS adopters, we also asked about what adaptations they had made to the program or its delivery to better engage communities who are underserved. During COVID-19, we added questions to make sure tele-delivery and distance training were feasible and accessible.

Due to COVID-19 onset, in April 2020 we revised the interview guide to capture additional data on the altered community context and “telePEARLS” delivery *via* phone or video-conferencing and invited Winter 2020 interview participants to do a follow-up interview. Interviews were scheduled for 30 min, and participants were provided a $50 incentive for participation. Organizations could choose how many people to invite to the interview; we conducted both individual and group interviews. Following each interview, we sent a brief quantitative survey to systematically capture data on participant demographics and professional background.

### 2.4. Data analysis

Data were stored and managed in REDCap (40) and Excel. We used the rapid framework method (41) to thematically analyze (42) interview data. These analytic approaches are appropriate for answering our research questions, highlighting similarities and differences across participants and generating unanticipated insights during rapidly changing pandemic times. Interview recordings were transcribed for analysis. We (LS, AP, MK) reviewed a sample of transcripts to generate initial codes (both deductive from the interview guide and inductive from emerging themes) to categorize the data, refining the codebook as needed in subsequent transcripts. We systematically reduced the data from original accounts into a coding matrix of codes by interviews, with data including participants' words, framing and illustrative quotes. For the last step, interpretation, we reviewed the matrix to make connections within and between participants and codes, and moved beyond individual case description to develop themes that provide possible explanations for what is happening in the data. These interpretation memos served as our preliminary findings which were refined into our results section with our study team and community partners.

These methods align with well-established trustworthiness criteria (43) to ensure rigor in our analysis. For credibility, or goodness of fit between participants' perspectives and how we represented them, we used prolonged engagement with the data and research triangulation with multiple members of our team including PEARLS organization staff as co-authors. For transferability, we will provide descriptions of study participants, their organizations, and their perspectives through illustrative quotes so that readers can determine whether our findings would be applicable to their context. We documented decisions made throughout the study for dependability and confirmability.

### 2.5. Designing equity—centered dissemination and implementation strategies

Formative research findings will be used to create new PEARLS dissemination and implementation (D&I) strategies that center the strengths and needs of communities who are underserved. Dissemination strategies aim to change attitudes about and increase awareness, knowledge of, and intention to adopt EBPs like PEARLS through messaging and channels designed for organizations that engage these older communities. Implementation strategies aim to build capacity for selecting, adapting, and integrating PEARLS into these delivery settings and support systems (44). We shared study findings with our Community Advisory Board, Scientific Advisory Board, and internal and external communication experts to co-develop these new D&I strategies.

## 3. Results

We approached 45 organizations *via* email and phone through state and local networks and through snowball sampling; 24 agreed to participate. Reasons for not participating from the other 21 organizations included declined (e.g., too busy, not enough staff capacity to participate; *N* = 7) and were not able to be contacted (*N* = 14). While data saturation can be achieved at 12 interviews (45), we engaged additional participants for variation in geographic area, organization types, PEARLS adoption status, and populations served.

We conducted 39 in-depth interviews with 24 organizations in 2020. Sixteen interviews were conducted between February and March (before COVID-19 pandemic) and 23 interviews were conducted between July and September (during the COVID-19 pandemic). Fourteen (61%) of these were follow-up interviews with organizations interviewed pre-COVID-19. Interviews lasted mean (SD) 56 (16) min (range 27–85 min) and included 1–5 participants. Interview participant characteristics are provided in Table 2. Interviewees were middle-aged and older adults, 60% female, and 43% communities of color. Most participants had worked at their organization (81%) and had been in their role (68%) for five or more years.

**Table 2:** Characteristics of interview participants (*N* = 37)^a^.

**Respondents**	***n***	**%**
**Age**		
< 30	1	2.70
31–40	9	24.32
41–50	5	13.51
51–60	10	27.03
61–70	6	16.22
71–80	1	2.70
Missing	5	13.51
**Gender**		
Female	22	59.50
Male	14	37.83
Did not specify gender	1	2.70
**Race**		
American Indian/Alaskan Native	1	2.70
Asian American/Pacific Islander	11	29.73
Black or African American	2	5.41
White	21	56.76
Did not specify race	2	5.40
**Ethnicity**		
Latino	7	18.92
Not Latino	30	81.08
**Profession** ^*^		
Social work	22	59.46
Gerontology/aging	14	37.84
Behavioral/mental health	10	27.03
Administration	7	18.92
Public health	3	8.11
Health care	2	5.41
Other professions^b^	8	21.62
Missing	1	2.70
**Education**		
Some college	3	8.11
College graduate	16	43.24
Post college/graduate school	17	45.95
Missing	1	2.70
**Length at organization**		
1–2 years	2	5.41
3–4 years	5	13.51
5+ years	30	81.08
**Length in role**		
1–2 years	7	20.00
3–4 years	5	13.51
5+ years	25	67.57

a Two of 39 interview participants did not report survey data.

b Other professions includes Communications, Sociology, College, and Student Services.

* Participants could check all that apply, so total sums to more than 100%.

Table 3 shows the attributes of the organizations that participated in the interviews, all of which served older adults experiencing poverty. Half (50%) were community-based social service organizations, 30% were potential funding organizations, and 10% were other partners. Organizations were split between current PEARLS adopters and potential PEARLS adopters (organizations not currently delivering PEARLS). Almost all (92%) were engaging older communities of color (70% Latino, 58% Asian, 21% Black, and 4% Indigenous), and older adults who spoke languages other than English (70% Spanish, 38% Chinese, and other languages including Korean, Vietnamese, Japanese, Hmong, Khmer, Tagalog, Arabic, Russian, Ukrainian, and Asian and Indigenous languages that were not specified). Fifty-eight percent of organizations served rural areas. Interviewees represented a mix of roles −83% of organizations interviewed had a decision-maker and 58% of organizations had a front-line staffer participate.

**Table 3:** Participating organizations that reach older adults who are underserved in Washington and California (*N* = 24).

**Organization**	***n***	**%**
**State**		
California	13	54.17
Washington	11	45.83
**Type of organization** ^*^		
Funder	7	29.17
CBO	15	62.50
Other partners	7	29.17
Food access	3	12.50
Community mental health	2	8.33
Faith-based organization	2	8.33
State	2	8.33
**Population served by CBO** ^*^		
Communities of color	22	91.67
Black	5	20.83
American Indian/Alaskan	10	15.87
Native		
Asian	7	11.11
Latino	3	4.76
Non-English language preferred	2	3.17
Rural	8	12.70

* Participants could check all that apply, so total sums to more than 100%.

Our formative research focused on three central questions: (1) What are the needs and priorities of older adults in communities who are underserved by social and health resources? (2) What are the needs and priorities of organizations that engage these communities? and (3) What are the most important strategies, collaborations, and adaptations needed for adopting and delivering PEARLS in communities underserved by social and health resources?

### 3.1. Priorities and needs of older adults in communities who are underserved

#### 3.1.1. Older adults living in poverty remain underserved by mental health and health care

Interviewees highlighted how older adults experiencing poverty remain underserved by mental health and health care. Despite being connected to home and community-based services, these supports were insufficient or inappropriate for meeting older adults' array of health needs. In addition to health care, older adults also required assistance with basic needs like food, housing, and heating their homes during extreme weather. Participants shared that ageism and social isolation due to poverty, mobility limitations, lack of transportation, loss of function, or caregiving duties, meant that older adults' needs remained unmet. As one potential adopting CBO in California put it, “*The number one thing is access to care….they are simply not being seen, it's like they are invisible* (WA014).”

#### 3.1.2. These older adults continue to experience isolation, depression, and barriers to care

Isolation and depression are ongoing issues for the older adults in communities served by the organizations we interviewed. Participants described how the older adults they engage have faced years of adversity and the many changes that come with aging. As one current CBO adopter in Washington stated: “*Because if you've lived on the planet for a long time, you have lost a life. Some clients are so depressed because they've had massive things that's changed* (WA003).” Cultural barriers and social stigma also make it challenging for older adults to openly discuss feelings of depression. Older adults with depression will often describe it *via* symptoms or feelings, such as feeling lonely, sad, worried, frustrated, stressed, anxious, down, experiencing chronic sorrow, too many problems, or needing social support. Given this challenging context of isolation, adversity and stigma, any strategy for community providers to address depression must start with building trust to engage older adults in care.

#### 3.1.3. The pandemic has aggravated unmet health and mental health needs for older adults

The COVID-19 pandemic exacerbated these needs into a “*life or death situation*” (current PEARLS adopter in California, CA005), as social distancing and fears of contagion made it challenging for older adults to access services: “…*clients do not want people coming in [to their homes] even though they want to see somebody*.” (CA026, food access organization in California). For cultures for whom community is an essential value, the social isolation has been devastating: one Washington funder shared the impact on Indigenous communities: “*We don't know how to be apart from each other. We don't know how not to share everything we have with each other. We don't know how not to gather for our dances, for our ceremonies, for our language. We don't know how not to do those things, and it's really hurting people* (WA014).”

#### 3.1.4. Help-seeking and tele-health care has shifted during the COVID-19 pandemic

While some organizations felt older adults have been even more cautious about seeking assistance during the pandemic, others felt the acuteness of need has made older adults more willing to ask for help. Organizations have pivoted to remote service delivery during COVID-19, though both providers and older adults prefer receiving services in-person when they can do so safely. There were mixed opinions about remote service delivery from both current and potential adopters—some organizations have seen increased access to services that no longer have to rely on transportation, and appreciate having a service to connect with older adults who are isolated and unable to access resources. Other interview participants called out the challenges in access and privacy with tele-care for both older adults and staff from priority communities who have been underserved: “*I mean, people may have those phones, but I know there's a lot of people who still aren't comfortable if they don't have a smartphone… in a lot of the rural areas, we have problems even with our own staff being able to get on VPN and get access and keep access* (WA013).”

### 3.2. Priorities and needs of organizations that engage older adults in communities who are underserved

#### 3.2.1. The recent social context has made it harder for organizations to engage older adults who are underserved

In addition to providing social care and linkages to what health care is available and accessible to their communities, CBOs were providing some mental supports to older adults. Those who have not yet adopted PEARLS did not feel equipped to address the levels of depression in their communities. The pandemic and other social challenges beyond COVID-19—a combative presidential election, police violence and continued racial injustice against Black and Brown communities, and extreme weather—have made older adults even more difficult to reach with services: one rural California organization (CA021) shared the need for “*increasing communication and provision so that people access mental health and behavioral health services during this time where the needs seem to be going up and suicide rates are going up, addiction is going up, and mental health crisis is going up. So we're continuing to provide resources, but gearing it a little bit more toward what's happening. It's just so many. It's not just COVID-19, but here in California, we have wildfires, we've had extreme heat, and we've had in the cities and even in the small towns, we've had protests and civil unrest because of racial injustice*.”

#### 3.2.2. Organizations require more training and capacity for staff to provide depression care

Staff at CBOs and funders that have not yet adopted PEARLS do not typically have mental health training and are cautious about addressing the topic with their clients particularly when they do not provide care or have appropriate services to refer them to. One California CBO serving older Latino and Chinese adults shared: *I know any staff that are non-licensed generally avoid using the term [depression]….My experience is that, I've often worked with frontline staff, degreed and non-degreed, that have this feeling that if they ask someone about depression, and they don't have a place to refer someone, then that's worse than if they didn't ask it at all* (CA026).” Organizations believe their staff need more and better training about the importance of addressing depression and how to recognize symptoms so that older adults can be connected with care. That said, most organizations shared that staff are beyond maximum capacity during the pandemic and struggling to do more with less.

#### 3.2.3. Organizations identify new services based on community needs and networks

In terms of how organizations identify services to support their older communities, funders plan new programs based on community needs, and hear about new programs through professional networks. Community-based organizations and other partner organizations also learn about new programs through networks (e.g., peer networks such as local coalitions, partner organizations for referrals and funding, and health fairs) though this learning happens more organically than actively seeking out new services as “*every day changes…so all information is good information* (WA016).” Once organizations hear about a program, they may look up further details using the Internet or print materials, websites, or brochures, but much of program's credibility is established through recommendations from peers or word-of-mouth. As one California potential adopter from a faith-based, food access organization shared, “*I listen to the networks that we're involved in to see who might be doing that, …. and be able to get information from colleagues about who's done this program, who knows about this program, and is going to talk to me about their experience with the program* (CA027).” For interview participants who are front-line providers or managers at large organizations, they sometimes don't have a say in what new services they adopt but rather are told by leadership.

#### 3.2.4. Organizations desire sustainable, accessible programs that align with cultural values

Organizations look for programs with cultural flexibility, stable funding, and accessible training that inspire commitment from their staff, and that fit with both their organizational culture and values and those of the communities they serve. Cultural flexibility means the program can adapt to accommodate different cultural norms, values and beliefs, and has a history with marginalized groups, which is not typically how evidence-based programs are perceived. There were mixed opinions about programs being “evidence-based”—funders tended to value this as an indicator of quality and access to funding, whereas CBOs were mixed. Some CBOs feel evidence-based program status is not important as it is meaningless to the community and what matters most is evidence created for the communities they serve. Other CBOs are actively interested in adopting evidence-based programs that have been shown to work for their communities because funders require this and they want some assurance the program will work if investing scarce resources. Given communities' limited access to services in resource-constrained environments in which organizations operate, there must be alignment between what communities' want and need, and the organization's ability to sustain services. As one potential adopter CBO in Washington shared: “*We want to make sure if we put something in place, a), we hear the voice of the community, and b) it's sustainable so it's not going to blow in the wind* (WA016)”.

### 3.3. Strategies, collaborations, and adaptations for delivering PEARLS with CBOs that engage communities who are underserved

#### 3.3.1. Train trusted staff from communities to improve access, delivery, and impact

Most interview participants strongly recommended that staff delivering PEARLS should be from the community being served, in order to best meet the needs of older adults. As one current PEARLS adopter described: “*We have a small team, three of them were born and raised in the community….They know the community. They understand how to talk to people that may not talk like them, but they understand it. It's important to be able to serve so that they don't feel like you're talking down to them* (WA003).” This was amplified during the COVID-19 pandemic when older adults were further isolated and engagement had to be done remotely.

#### 3.3.2. Programs must be culturally appropriate for both engagement and outcomes

Cultural appropriateness is also paramount given that many communities have experienced a long history of programs and services that are a cultural mismatch, potentially doing more harm than good. Some potential adopting organizations were cautious about whether PEARLS would be a good fit for their communities. One potential PEARLS adopter, a social service and food access organization in California, summed it up as follows: “*Oh, yes, we've heard of that [PEARLS]. I don't know if that really works for our clients. And I'll dig into that a little more. Some of our home-bound clients, that might be most isolated or most depressed, and very, very poor, extremely low income, just barely housed. I think there's a perception with some of my team, that programs like this aren't geared toward that population …. how effectively has it been offered and sustained in communities of color? (CA026).”*

#### 3.3.3. Clarify that quality depression care can be provided by non-clinical staff with clinical support

Many participants also voiced the perception that PEARLS coaches must have advanced educational degrees and be clinically trained and licensed, when in fact the model was designed to train front-line staff without these credentials to deliver mental health services. Likewise, participants believed that the clinical supervisor required to support PEARLS coaches was hard to access given clinical workforce shortages. As one potential adopting community-based organization in California shared: “*I think I could count on both hands how many psychiatrists we have available... We have maybe 10, right? That's serving all of [a rural] county and probably beyond and only a handful of those…take Medicare. So we have a huge shortage. It's really hard to retain doctors and specialists in this area* (CA020).”

#### 3.3.4. Funding plus other implementation supports remain key for equitable implementation

Funding was also seen as a challenge to PEARLS adoption, from both current and potential adopting organizations. Organizations use a variety of funding sources to support PEARLS, including redirecting organizational funding (rather than chasing new funding which can be a major time investment to secure). Funding is used not just for staff time and training to deliver care but also to do engage persons in care *via* outreach and referrals from internal and external partners, and evaluating impact and adapting as needed. While some CBOs are already connected with funding organizations, some funders desire help connecting and collaborating with CBOs who engage older adults living in underserved communities. CBOs want to partner with funders for financial support as well but find some of the pathways to funding too restrictive or complex: “*Department of Mental Health is huge. So, getting your foot in the door and getting connected is not an easy feat* (CA027; potential adopter CBO social service and food access organization in California).” While funding was important to launch the program, training and staffing were also key pieces of the adoption process. For example, having monthly group technical assistance calls with our center helped nurture a community of practice to support organizations to adapt, deliver, and sustain PEARLS.

#### 3.3.5. Organizations that have adopted PEARLS highlight fit with existing staff and community

For organizations with PEARLS programs, the decision to adopt the program had often been made by organization leadership based on perceived fit with staff and community needs and priorities. Funders and CBOs view the program as aligning with their mission and communities, and appreciate being able to integrate PEARLS into what they are already doing to support older adults. Staff shared how PEARLS' focus on problem-solving could help older adults from diverse cultural backgrounds address concrete causes and symptoms of depression right away. As one California community health center noted: “*Most of my staff felt PST [Problem-Solving Therapy] is much easier, because our patient population are not that comfortable to talk about feelings… So we help them to come up with a solution. This is more culturally relevant, or more culturally acceptable to them* (CA015).” Some CBOs noted that older adults are more comfortable talking about depression after completing PEARLS and seeing that it is possible to recover.

#### 3.3.6. PEARLS tools can also support self-care for front-line social service staff

Organizations currently doing PEARLS during COVID-19 highlighted how tools have helped support staff as well. One California funder who is a current PEARLS adopter shared: *We've talked a lot about self-care during this time. Aside from the COVID-19 aspect of everything and making sure you're washing your hands and all that stuff, really focusing and having them look at how are they taking care of themselves, which I know we already have to do in a helping profession, but now even more so our providers have gone from being a provider to being a provider while trying to be a teacher and do childcare and do... all of these multiple worlds are colliding at the same time, and that has been a struggle for a lot of folks. So making sure that they really are taking the time that they can to create that time, to carve that time out, to really make sure that they're just connecting, that they are finding good ways to take care of themselves. Just like they're walking their clients through doing those things, they need to be able to do that for themselves... I'm hoping it's been impactful and helpful for them. It's really hard to pour from an empty cup and it was really easy to get yourself drained during the last 3 months, if you didn't make a concerted effort to take care of yourself* (CA002).

#### 3.3.7. Adaptations are an important implementation strategy for health equity

Table 4 summarizes current adaptations that organizations have made to support PEARLS delivery with older adults who have traditionally been underserved, and recommended adaptations to better fit their organizations or community. These modifications include changes to distance training and remote delivery with the onset of COVID-19. As an implementation strategy for promoting health equity (47), partnering with organizations, staff, and older adults to adapt what, how and where PEARLS is being delivered can facilitate program implementation in populations who have been historically underserved.

**Table 4:** Current (C) and recommended (R) adaptations^a^ to PEARLS to engage older adults who are underserved.

**PEARLS**	**Adaptations**
**PEARLS content**	
English-language written materials	• Use terms and vocabulary in English and other languages that are culturally appropriate (C) • Translate materials into other languages (currently available in Spanish, Chinese, Russian, and Somali) (C) • Have PEARLS materials translated into additional languages (mixed opinions on whether this should be done by our center or by organization) (R)
Psychoeducation, PST, BA, linkages to community-clinical	• Additional supports tailored to their community that complement PEARLS (e.g., case management, motivational interviewing, mindfulness and relaxation) (C)
**Context (COVID-19) and PEARLS delivery**	
In-person engagement	• Engagement *via* phone or video-conferencing (C) • Take additional time/calls to listen and hear their story; it may be necessary to assist a new client with urgent needs (food, heat), before the focus on PEARLS (C) • Drop off food and forms masked and distanced to build rapport (C)
In-person delivery	• PEARLS delivery *via* phone or video-conferencing (C) • Many older adults do not have access to or comfort using video-conferencing that requires reliable internet, data plans, hardware (smartphone, tablet, and computer) (C) • Can be hard to remotely teach older clients to use tech (C) • Guidance on how to adjust PEARLS for remote delivery (R)
In-person assessment	• Mailed forms or dropped off at older adults' home, and split assessment into multiple calls (C) • Review and update enrollment paperwork given pandemic reality many older adults are experiencing (R)
Master's level social workers and nurses	• Community health workers, interns, case managers (high school/GED, bachelor's, or graduate) (C)
6–8 sessions (3 weekly, 2 biweekly, and 3 monthly)	• Extend frequency to 10-15 sessions (still time-limited but allow additional support for older adults with complex lives (especially during COVID-19) and ease transition from biweekly to monthly sessions (R)
Individual intervention (one-on-one)	• Include group component to strengthen social and peer support during and after program (R)
**Training and technical assistance (TA) strategies**	
One-time in-person training (2 days)	• Training done *via* recorded video demonstrations, quizzes, live Zoom teaching, practice, and feedback (C) • Include case studies and role-plays about engaging communities who are underserved and delivering PEARLS in different community and cultural contexts (R) • Provide booster trainings for CBOs that engage communities who are underserved (R)
Ongoing TA (phone, email, and video-conferencing)	• Monthly TA calls for trained organizations to foster community of practice (C) • Include more content specific to engaging underserved communities. (R) • Offer case review and questions with clinical supervisor (R)
**Implementation and funding**	
Outreach done by research staff with grant funding.	• Outreach done by CBOs that already engage communities who are underserved. Can be time-intensive and often not covered as part of program funding. (C) • More direct outreach to populations at-risk (R)
Funding provided through research grant	• Provide letters of support for funding and share resources during TA calls (C) • Support organizations to identify and secure funding (R)
Clinical supervision provided through grant	• Support organizations to arrange clinical supervision (R) • Clarify clinical supervisor roles, responsibilities, options (not just a psychiatrist) (R)

PST, Problem-Solving Treatment; BA, Behavioral Activation.

a Organized using Framework for Reporting Adaptations and Modifications to Evidence-based interventions (FRAME) (46).

### 3.4. Equity-centered dissemination and implementation strategies

**Figure 1:**
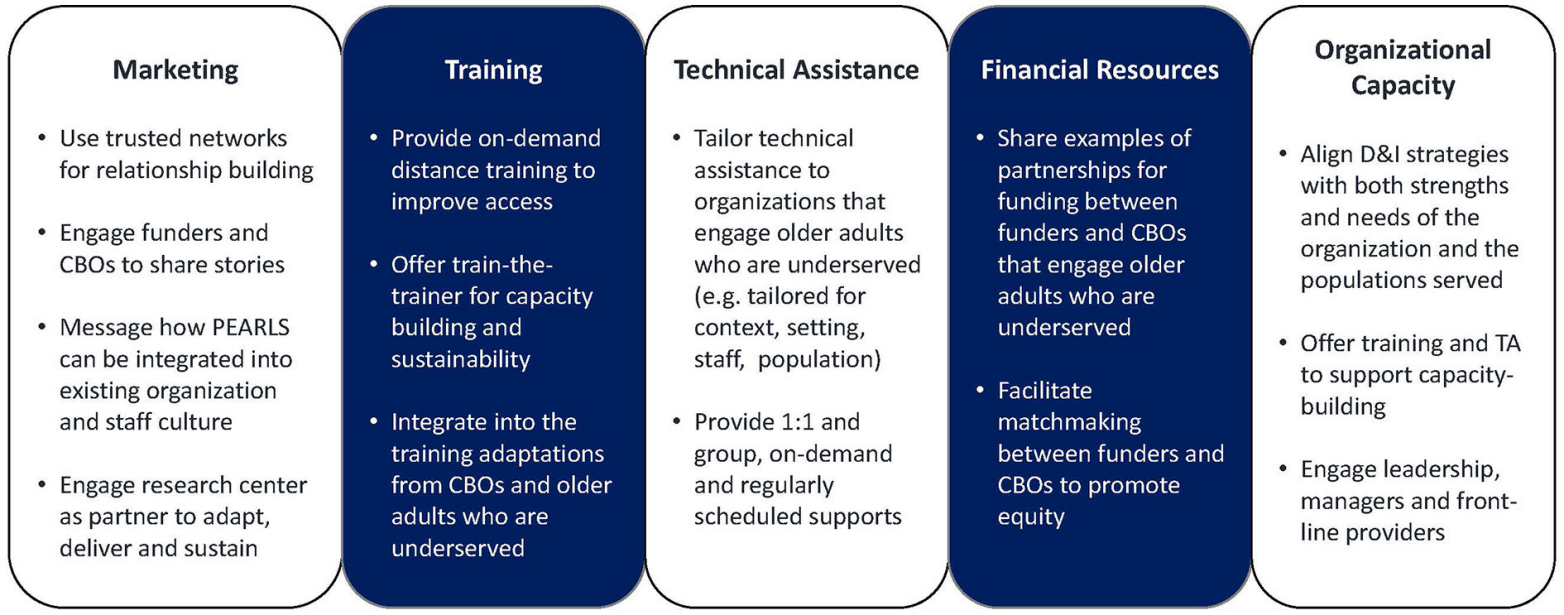
Adapted from knowledge to action (K2A) framework, translation supporting structuring, Wilson et al. (48).

These findings were used to create new PEARLS dissemination and implementation strategies that prioritized the strengths and needs of underserved communities and the organizations that engage them (Figure 1). For the dissemination strategies, our internal and external communication experts created new messaging to emphasize the ways in which PEARLS can work in partnership with communities and organizations, and clarify misconceptions about program accessibility, appropriateness, and cost. Messages were actively disseminated and tailored to different audiences (funders, CBOs, or other partner organizations) and delivered both *via* our center and peer organizations that have done PEARLS with older adults in our priority populations. Since we cannot use word-of-mouth locally (we are delivering this strategy remotely across two states), we are using written and verbal channels such as website, phone, email, and webinars to build relationships.

For the equity-centered implementation strategies, we are holding virtual community conversations and provide one-to-one support to organizations to engage partners, assess capacity, need, and PEARLS fit, and discuss what adaptations are appropriate and desired. These supports focus on what organizations shared are important to their staff and to their communities, such as broadening “depression” beyond clinical diagnoses and stigma to addressing loneliness, isolation and what matters to older adults. Implementation strategies will also emphasize stories from staff and older adults about PEARLS' impact; clarify that ongoing training and technical assistance are available for capacity-building; and share examples of how resource-constrained organizations have partnered for funding and clinical supervision.

## 4. Discussion

Community-based organizations (CBO) have provided essential support to older adults who are underserved, including meal delivery and access to COVID-19 testing, vaccines, and other health care. Isolation and depression have emerged as urgent issues among older adults and the CBOs that engage them, due to the pandemic's disproportionate impact on older communities, increased anxiety and fear, and decreased social and physical connections. When looking for new services and supports for older communities who are underserved, CBOs want programs that fit organization, staff and community strengths and needs, are culturally appropriate and flexible, have stable funding, and provide accessible training and capacity building. These findings align with similar formative research to identify strengths and needs of trusted community-based organizations as partners in evidence-based health promotion that reach community members who are often marginalized or stigmatized (29, 49, 50).

Our findings align with implementation science and practice recommendations to improve health equity. While programs like PEARLS have traditionally highlighted their effectiveness on clinical outcomes or being “evidence-based” (51, 52), the literature bridging cultural adaptations and implementation science (53) to reduce racial and ethnic disparities in mental health care emphasizes the importance of communicating about an intervention's “social validity” (51). Social validity is the acceptability and usefulness of a program which is influenced by one's worldview (e.g., stigma about depression), practical realities (e.g., caregiving duties, work), and access (or lack thereof) to resources such as to transportation, mental health insurance, and culturally and linguistically appropriate care. Improving and communicating about PEARLS social validity is thus essential for reducing disparities in older adults' access to depression care Furthermore, current and recommended adaptations to PEARLS and how it is delivered are a key implementation strategy for health equity (47): they center the CBOs that reach older populations most at “risk of risks” (54) to deliver quality care that is socially valid and fits different contexts, thus improving engagement, delivery and outcomes (52).

Our learnings from interviews support recommendations from social marketing and communications to facilitate organizational behavior change—the adoption of depression care by social service organizations—by tailoring and targeting messages to front-line staff, managers, and funders using narratives that resonate with their values and context (55). For instance, finding suggest messaging to front-line staff about how PEARLS can meet both them and their older adult clients where they are in their communities to reduce depression and isolation in ways that are accessible and appropriate, and providing training to support engaging older adults in depression care given stigma and history of injustice. Managers who are often tasked with both decision-making and doing would benefit from messaging about PEARLS flexibility and adaptability to support both their staff and their older adult communities, such as supporting staff self-care *via* clinical supervision and extending the number of sessions to support older adults with complex health and social needs. For funders who are increasingly called upon to address health inequities but without additional resources to do so, we can better communicate about how to use existing funding to fill gaps in care for older adults (e.g., Older Americans Act Title III-B and D funding); Medicaid funding such as Tailored Supports for Older Adults (TSOA), Medicaid Alternative Care (MAC), and COPES Ancillary; and the Mental Health Services Act Prevention and Early Intervention funding) and connecting them with local CBOs to reach older adults who have been underserved by depression care. Engaging these organizations as partners in both dissemination and implementation research and practice further bridges research to practice (56), centering their wisdom about how to adopt, adapt, deliver, and sustain PEARLS for improving equitable access to depression care.

The strengths of this study are using qualitative research methods and a social marketing approach to learn from CBOs with community wisdom to design an intervention to better support their adoption of quality depression care. This study aligns with recent calls to center equity in implementation science so that these strategies close rather than widen gaps for older communities who are underserved. These recommendations include focusing on reach from the very beginning, designing interventions for these populations and resource-constrained settings with implementation in mind, creating dissemination and implementation strategies that address inequities in access to care, understanding what can be adapted to better meet organizations' and communities' needs while maintaining program fidelity, and using an equity lens for evaluating how well and how much the intervention is working (47).

However, this research comes with several limitations. First, data were collected right as the pandemic was emerging and in its 1st year. Current partnerships with CBOs suggest that many organizations are still focusing on addressing basic needs of older adults and wanting to address inequities in access to care while they contend with economic challenges. Second, organizations who were willing and able to participate in this research may not reflect all organizations that reach older adults who are underserved, nor are all older adults who are underserved represented in this research. Third, most interview participants had a college education or more and had worked at their organization and in their role for 5 years or more. While being more educated and having a longer tenure at their organization may have provided advanced skills and deeper knowledge of both their organization and community, findings may not reflect the perspectives of front-line staff with less formal education or newer to their position or organization. Lastly, we recognize that our proposed organizational intervention and the one-one-one PEARLS program cannot fully eliminate the social determinants of health and the historical and contemporary injustices that have created older adult health disparities. Policy, systems and environmental changes and other structural interventions are needed to address these drivers of inequities in late-life depression burden (57).

In closing, this study describes formative research with organizations who are engaging older adults experiencing poverty and are underserved by depression care: older adults of color, who are linguistically diverse, and/or live in rural areas. Findings highlight how these older adults remain underserved by mental health, health and social care, which intensifies the burden of depression and isolation. The COVID-19 pandemic exacerbated these needs and also created opportunities with normalizing both help-seeking (through shared experiences, conversation and empathy about feeling depressed, anxious or traumatized) and remote care delivery (given in-person delivery was not a viable option). Organizations that engage older adults underserved by depression care see challenges given stigma, acute pandemic and environmental stressors, chronic injustices and resource scarcity, yet recognize their role to connect marginalized older adults to better care. Existing networks can be tapped to raise PEARLS awareness as one in-house solution for address inequities in access to depression care, aligning with organization's needs, preference, and values for programs that are person-centered and culturally appropriate, and have stable funding, accessible training, and flexibility to fit the culture of their organization and older adult communities. These findings guided new equity-centered dissemination and implementation strategies to better engage and support organizations who reach older adults who are underserved as depression care providers. We are currently partnering with organizations in California and Washington to evaluate whether and how these D&I strategies increase equitable access to PEARLS and plan to share findings in 2024.

## Data availability statement

The raw data supporting the conclusions of this article will be made available by the authors, without undue reservation.

## Ethics statement

The studies involving human participants were reviewed and approved by University of Washington IRB. Written informed consent for participation was not required for this study in accordance with the national legislation and the institutional requirements.

## Author contributions

LS conceived of the study, interviewed participants, co-led the analysis, and drafted the manuscript. AP made intellectual contributions to study development, interviewed participants, co-led the analysis, manuscript review and editing, and approved the final version. MK made intellectual contributions to study development, participated in the analysis, manuscript review and editing, and approved the final version. BB, PH, and MS made intellectual contributions to study development, manuscript review and editing, and approved the final version. KH-H and SW contributed to manuscript development, review and editing, and approved the final version. SI and LB participated in data collection, contributed to manuscript review and editing, and approved the final version. All authors contributed to the article and approved the submitted version.
