# Supplementary material for: Partnering with community-based organizations to improve equitable access to depression care for underserved older adults in the U.S.: Qualitative formative research
Source: Front Public Health. 2023 Jan 30;10:1079082. doi: 10.3389/fpubh.2022.1079082 (PMC9922751; doi:10.3389/fpubh.2022.1079082)
Supplement: Supplementary file 1 [file Table_1.docx]

**Supplemental Table 1. Summary of formative research findings.**

| **Priorities and needs of older adults in communities who are underserved** | **Priorities and needs of organizations that engage these older adults** | **Strategies, collaborations, and adaptations to improve equitable access to depression care** |
| --- | --- | --- |
| - Older adults experiencing poverty remain underserved by mental health, health, and social care - Ageism, poverty, and stigma create barriers for talking about depression and accessing care - Depression and isolation are major issues for older adults who are underserved and orgs that engage them - Depression is described as symptoms or feelings (e.g., stress, loneliness) - COVID-19 and other 2020 crises exacerbated needs and isolation, and created barriers to access in-person services - During COVID-19, some older adults have been more cautious about seeking care, while others are more willing to seek care given acute need - Remote service delivery both helped and hindered access | - Organizations are providing social care, links to health care, and some mental health care - Organizations and staff do not feel equipped to address levels of depression in older communities especially during COVID where stressors have been exacerbated for both older adults and staff - Staff often do not have mental health training and are cautious about discussing depression without accessible referrals - Organizations would like more training for staff on recognizing depression to connect older adults to appropriate care - Organizations learn about new programs from various networks - Organizations look for programs with cultural flexibility, stable funding, accessible training, and that fit with both organizational culture and values and those of the communities they serve - Mixed opinions about value of “evidence-based programs” | - Staff that provide depression care should be from communities being served to improve engagement and trust - Cultural appropriateness is key to improve engagement and outcomes – need to know program is a good fit given long history of cultural mismatch - Belief that depression care staffing requires clinical credentials and lack of workforce with these - Need funding to adopt new services – preference for diverse funding given resource scarcity - Opportunities for funders and CBOs to partner to improve access; though some funding comes with too many strings - Adopters decided to do PEARLS based on fit with staff and community priorities and needs - PEARLS tools may support both staff and older adults - PEARLS adaptations welcome as an implementation strategy for health equity |
